# Ionic Liquid-Based Centrifuge-Less Cloud Point Extraction of a Copper(II)–4-Nitrocatechol Complex and Its Analytical Application

**DOI:** 10.3390/molecules30153287

**Published:** 2025-08-06

**Authors:** Denitsa Kiradzhiyska, Nikolina Milcheva, Miglena Ruzmanova, Fatma Genç, Petya Racheva, Kiril Gavazov

**Affiliations:** 1 Research Institute at the Medical University of Plovdiv, 15A, Vassil Aprilov Blvd., 4002 Plovdiv, Bulgaria; denitsa.kiradzhiyska@mu-plovdiv.bg (D.K.); nikolina.milcheva@mu-plovdiv.bg (N.M.); 2 Department of Chemical Sciences, Faculty of Pharmacy, Medical University of Plovdiv, 120 Buxton Bros Str., 4004 Plovdiv, Bulgaria; miglena.ruzmanova@mu-plovdiv.bg (M.R.); petya.racheva@mu-plovdiv.bg (P.R.); 3 Faculty of Pharmacy, Istanbul Yeni Yuzyil University, 26 Yılanlı Ayazma Caddesi, Istanbul 34010, Turkey; ftmgenc@yahoo.com

**Keywords:** copper(II), mixed micelle-mediated system, Aliquat 336, 1,2-dihydroxy-4-nitrobenzene, centrifuge-less cloud point extraction, spectrophotometric determination

## Abstract

A novel centrifuge-less cloud point extraction (CL-CPE) method was developed for the spectrophotometric determination of copper(II) using 4-nitrocatechol (4NC) as the chelating agent. The extraction system utilizes a mixed micellar phase composed of the nonionic surfactant Triton X-114 and the ionic liquid (IL) Aliquat^®^ 336 (A336). The extracted ternary ion-association complex, identified as (A336^+^)_2_[Cu(4NC)_2_], exhibits a maximum absorbance at 451 nm, with a molar absorption coefficient of 8.9 × 10^4^ M^−1^ cm^−1^ and a Sandell’s sensitivity of 0.71 ng cm^−2^. The method demonstrates a linear response in the copper(II) concentration range of 32–763 ng mL^−1^ and a limit of detection of 9.7 ng mL^−1^. The logarithmic extraction constant (log *K*_ex_) was determined to be 7.9, indicating efficient extraction. Method performance, evaluated by the Blue Applicability Grade Index (BAGI) and the Click Analytical Chemistry Index (CACI), confirmed its feasibility, practicality, simplicity, convenience, cost-effectiveness, environmental friendliness, and analytical competitiveness. The proposed IL-CL-CPE method was successfully applied to the analysis of a dietary supplement, a solution for infusion, and synthetic mixtures simulating various copper alloys.

## 1. Introduction

Copper is a highly conductive, malleable, and ductile metal that plays a central role in numerous industrial and technological applications. It is the second most widely used non-ferrous metal [1] and ranks as the 26th most abundant element in the Earth’s lithosphere [2]. Of the 808 identified copper-bearing minerals [3], sulfide and oxide forms hold the greatest economic importance [4]. Extracting copper from these sources involves complex, multi-stage operations that yield significant amounts of slag, dust, and aerosols, which, if not properly managed, can pose serious environmental and health risks [1,5,6].

Despite its potential toxicity at high doses, with an upper tolerable intake level of 10 mg per day [7], copper is classified as an essential trace element necessary for nearly all living organisms. It is involved in numerous biochemical reactions and functions as a cofactor in various enzymes. In the human body, copper is primarily stored in the liver, muscles, and brain, with total body levels typically ranging between 90 and 110 mg [8]. The European Food Safety Authority recommends a daily intake of 1.3 mg for women and 1.6 mg for men aged 18 and older [9].

Notably, copper deficiency has become more recognized and better characterized than in the past [10]. This condition has been linked to a range of health issues, including hematological abnormalities, skeletal deformities, neurological disorders, and impaired immune function [7,10,11].

A variety of spectroscopic techniques have been employed to determine copper. These include atomic absorption spectrometry, inductively coupled plasma optical emission spectrometry, inductively coupled plasma mass spectrometry, and ultraviolet–visible spectroscopy (UV/Vis). Among these, UV/Vis stands out as a simple, cost-effective, rapid, energy-efficient, and widely accessible analytical method. It is frequently used in both research and quality control laboratories and can be readily coupled with various pre-concentration techniques, including solid-phase extraction [12], liquid–liquid extraction [13,14,15], and cloud point extraction (CPE) [16,17,18,19,20,21,22,23,24,25,26,27,28,29,30,31,32].

In recent years, CPE has emerged as a preferred method for the preconcentration of copper ions [16,17,18,19,20,21,22,23,24,25,26,27,28,29,30,31,32,33,34,35,36,37,38,39,40,41,42,43,44] and a viable option for routine analytical applications, primarily due to its environmental sustainability and cost-effectiveness. First introduced in the 1970s [45], CPE operates by heating a solution containing a nonionic surfactant to its cloud point, the temperature at which the homogeneous solution separates into two distinct phases. This phase separation is typically enhanced through centrifugation, followed by cooling. Hydrophobic species, such as neutral chelates and ion associates, tend to partition into the denser surfactant-rich phase (SRP), enabling efficient preconcentration before subsequent analytical determination.

Over time, numerous advanced variations of conventional CPE have been developed to improve performance and address specific limitations. These include microwave-assisted CPE (MA-CPE), ultrasound-assisted CPE (UA-CPE), room-temperature CPE (RT-CPE), mixed micelle-mediated CPE (MM-CPE), micro-CPE (M-CPE), ionic liquid-based CPE (IL-CPE), rapidly synergistic CPE (RS-CPE), centrifuge-less CPE (CL-CPE), and supramolecular solvent-based CPE (SUPRAS-CPE), among others. Each of these variants offers distinct advantages, such as reduced extraction time, improved selectivity, enhanced energy efficiency, lower operational costs, superior extraction efficiency, and greater procedural simplicity [46,47,48,49,50].

The objective of this study was to develop a novel CPE–UV/Vis method for the determination of Cu^II^, integrating the key advantages of CL-CPE, MM-CPE, and IL-CPE. The chelating agent selected for this work was 4-nitrocatechol (4NC), a well-established, commercially available compound that has recently been employed in our laboratory for the CPE–UV/Vis determination of Mo^VI^ [51] and V^V^ [52]. According to the Classification, Labelling and Packaging (CLP) regulation, 4NC is classified as a non-hazardous substance and does not require hazard pictograms, hazard statements, precautionary statements, or signal words on its labeling [53].

It is well established that in alkaline media, 4NC forms a stable anionic complex with Cu^II^, [CuL_2_]^2−^ [54,55,56]. However, this complex is unstable at pH values below 8.2 [54], which hampers its application in spectrophotometric determinations due to its high blank absorbance. This limitation can be overcome by introducing a cationic reagent capable of forming an electroneutral ion-association complex with the anionic Cu^II^–4NC species, thereby facilitating its extraction. The formation of such a ternary complex may shift the optimal extraction pH to lower values, favoring the prevalence of protonated the 4NC form (H_2_L; p*K*_H2L_ ≈ 6.7 [56]), which is essential for minimizing blank absorbance and improving analytical reliability.

In the preliminary phase of this study, two cationic ion-association reagents, benzalkonium chloride and Aliquat 336 (A336), were evaluated in conjunction with the nonionic surfactant Triton X-114 (TX). A336 demonstrated superior performance and was therefore selected for further method development. As a room-temperature IL with surfactant-like behavior under certain conditions, A336 forms a mixed micellar system with TX [50,51,57], which enhances the extractability of the Cu^II^ complex. This combination facilitates the complex formation and efficient extraction in a mildly acidic medium (optimal pH < p*K*ₐ of 4NC), thereby improving selectivity and reducing blank absorbance. A key advantage of TX over other nonionic surfactants, which accounts for its widespread use, is its low cloud point temperature. This feature has the potential to minimize energy consumption and reduce the risk of thermal degradation of analytes [50].

## 2. Results and Discussion

### 2.1. Optimal Operating Conditions

**Figure 1:**
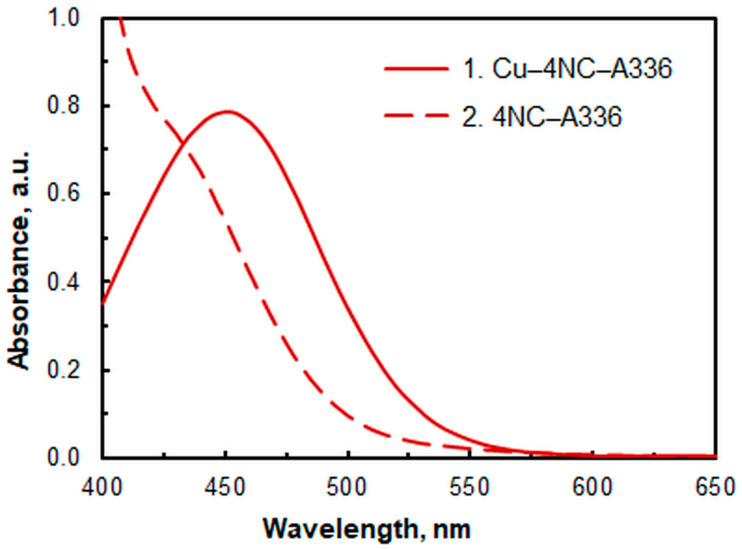
Absorption spectra of the Cu^II^–4NC–A336 complex against blank (1) and the blank against water (2). *c*_Cu_ = 8.8 × 10^−6^ M, pH = 6, *c*_NaNO3_ = 2.6 × 10^−2^, *w*_TX_ = 0.5%, *c*_A336_ = 3 × 10^−4^ M, *c*_4NC_ = 1.5 × 10^−4^ M, *t*_inc_ = 55 min.

The extracted ternary complex exhibits an orange color, with a maximum absorbance (λ_max_) at 451 nm, whereas the blank appears yellow. Figure 1 shows the corresponding UV/Vis spectra: (1) the spectrum of the complex against the blank, and (2) the spectrum of the blank against water.

**Figure 2:**
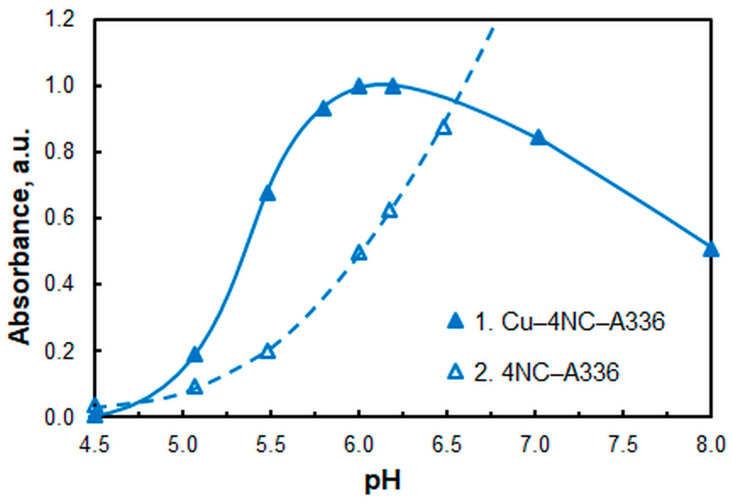
Effect of pH. *c*_Cu_ = 1.1 × 10^−5^ M, *c*_NaNO3_ = 2.6 × 10^−2^, *c*_A336_ = 3 × 10^−4^ M, *w*_TX_ = 0.5%, *c*_4NC_ = 1.5 × 10^−4^ M, *t*_inc_ = 55 min, λ = 451 nm.

Buffer solutions, prepared from 2 M CH_3_COOH and NH_3_, were used to study the effect of pH. As illustrated in Figure 2, the analytical signal achieves its maximum level within the pH range of 6.0–6.1. Subsequent studies were conducted at pH 6, as the blank absorption is lower.

**Figure 3:**
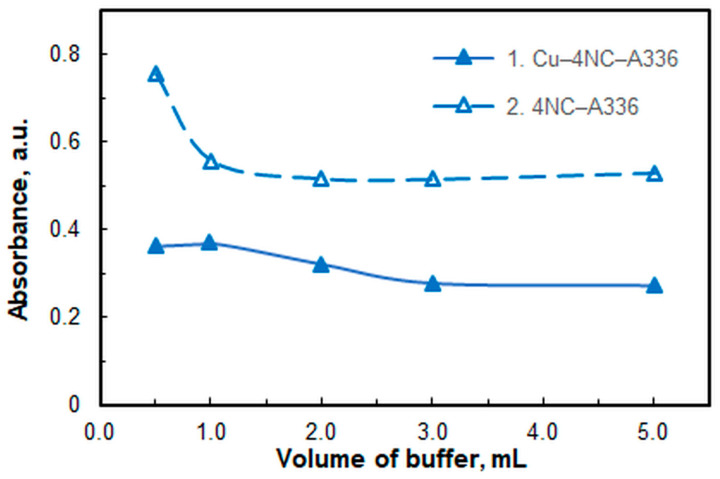
Effect of buffer volume (pH 6). *c*_Cu_ = 4.2 × 10^−6^ M, *c*_NaNO3_ = 2.6 × 10^−2^, *c*_A336_ = 3 × 10^−4^ M, *w*_TX_ = 0.5%, *c*_4NC_ = 1.5 × 10^−4^ M, *t*_inc_ = 55 min, λ = 451 nm.

The volume of the buffer solution (*V*_buff_) was also optimized (Figure 3). Subsequent studies were conducted in the presence of 1 mL of this solution. As can be seen at larger buffer volumes, absorbance decreases, which is likely due to competing complex formation with the buffer components. Centrifugation is necessary for phase separation when using buffer solution volumes smaller than 0.5 mL.

**Figure 4:**
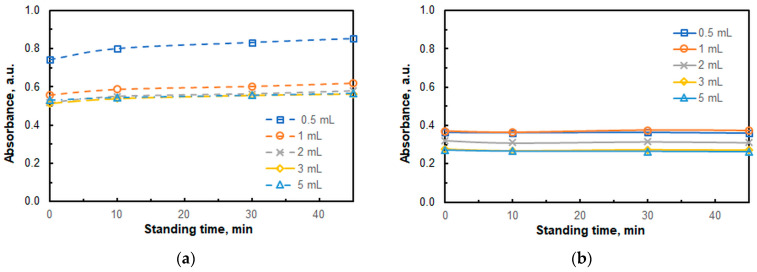
The combined effect of the buffer volume (pH 6) and standing time on the absorbance of the blank against water (**a**) and the absorbance of the Cu–4NC–A336 complex against the blank (**b**). *c*_Cu_ = 4.2 × 10^−6^ M (**b**), *c*_NaNO3_ = 2.6 × 10^−2^ (**a**,**b**), *c*_A336_ = 3 × 10^−4^ M (**a**,**b**), *w*_TX_ = 0.5% (**a**,**b**), *c*_4NC_ = 1.5 × 10^−4^ M (**a**,**b**), *t*_inc_ = 55 min (**a**,**b**), λ = 451 nm (**a**,**b**).

Figure 4 shows the results of the combined effects of two factors: buffer volume and standing time. The absorbance of the blank at *λ* = 451 nm increases over time, with the most pronounced effect occurring during the first 10 min (Figure 4a). This increase can be explained by a reaction in which the neutral form of 4NC (H_2_L) converts into an ion pair containing HL^−^ (Equation (1)):

H_2_L + A336^+^ º [(A336^+^)(HL^−^)] + H^+^ (1)

As demonstrated in Figure 3 and Figure 4a, the blank’s absorbance decreases in the presence of significant amounts of buffer. This is likely due to the suppression of the process shown in Equation (1). The same process also occurs in the sample containing Cu^II^. As the 4NC concentration significantly exceeds the Cu^II^ concentration, the resulting curves shown in Figure 4b are nearly flat and parallel to the *x*-axis.

**Figure 5:**
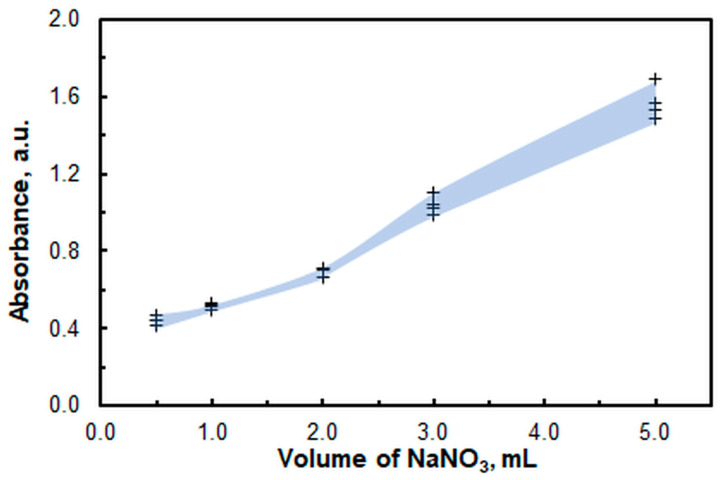
Effect of the NaNO_3_ volume (*c* = 1.3 M) on the absorbance of the blank (*n* = 4). pH = 6, *c*_A336_ = 3 × 10^−4^ M, *w*_TX_ = 0.5%, *c*_4NC_ = 1.5 × 10^−4^ M, *t*_inc_ = 55 min, λ = 451 nm.

Without a salting-out agent, the absorbance of the blank is unstable, even with a buffer present. Several salts were tested as stabilizers to reduce the standard deviation of the blank: NaCl, NaNO_3_, and K_2_SO_4_. The best results were obtained using NaNO_3_ (*c* = 1.3 M). The optimal volume for minimal standard deviation was determined to be 1 mL (Figure 5).

**Figure 6:**
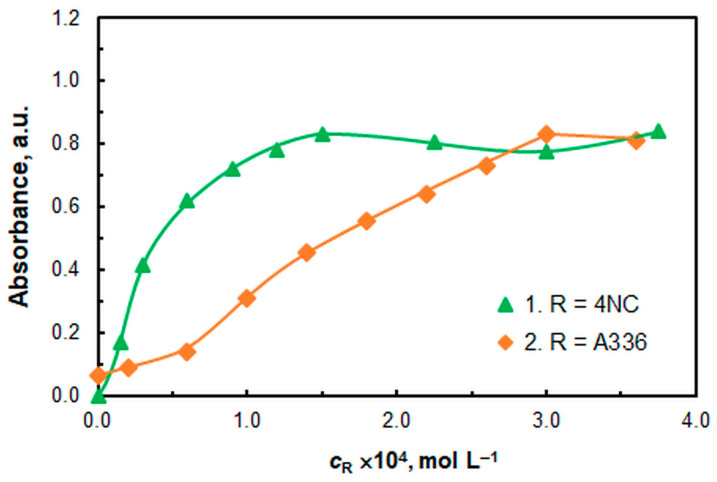
Effect of reagent concentrations: 4NC (curve 1) and A336 (curve 2). *c*_Cu_ = 9.4 × 10^−6^ M, pH = 6, *w*_TX_ = 0.5%, *t*_inc_ = 55 min, λ = 451 nm. 1—*c*_A336_ = 3 × 10^−4^ M; 2—*c*_4NC_ = 1.5 × 10^−4^ M.

Figure 6 shows the effects of the 4NC and A336 concentrations. The optimal 4NC concentration was found to be 1.5 × 10^−4^ M (curve 1). This value is five times lower than the concentration required for vanadium (V) determination [52]. At the current price of EUR 73 per 5 g, one package of this reagent would be sufficient for approximately 3700 samples, bringing the cost of 4NC per sample below EUR 0.020.

The cost of A336 is also reasonable (EUR 90.40 per 250 milliliters). Under the optimal concentration of 3 × 10^−4^ M (Figure 6, curve 2), a single 250 mL package would be sufficient for approximately 40,000 samples.

It is noteworthy that at *c*_A336_ = 0, the resultant absorbance is >0. The most probable explanation for this phenomenon is the partial extraction of the electrically neutral [Cu(4NC)] complex, which has been reported in some studies [54,56,58].

**Figure 7:**
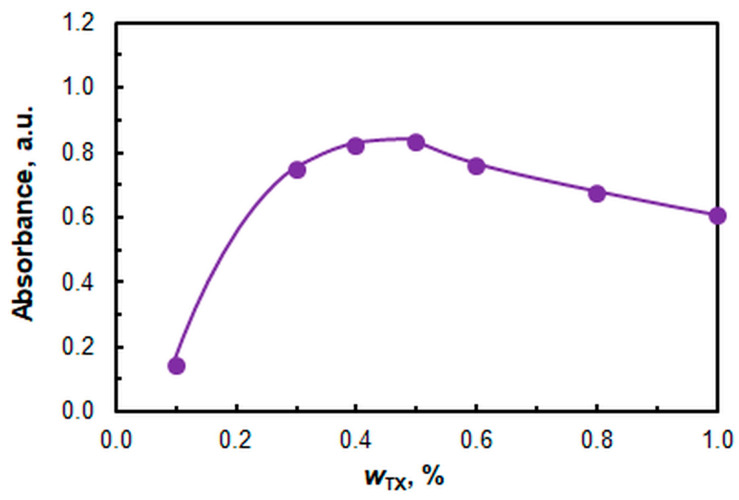
Effect of TX mass fraction. *c*_Cu_ = 9.4 × 10^−6^ M, pH = 6, *c*_A336_ = 3 × 10^−4^ M, *c*_4NC_ = 1.5 × 10^−4^ M, *t*_inc_ = 55 min, λ = 451 nm.

The effect of the nonionic surfactant mass fraction (*w*_TX_) is displayed in Figure 7. All subsequent studies were conducted at a *w*_TX_ = 0.5%. Assuming that the average molar masses of TX and A336 are 537 g M^−1^ [59] and 432 g M^−1^ [60], respectively, it can be calculated that the molar ratio between the two surfactants (*n*_TX_:*n*_A336_) under optimal conditions is 31, and the corresponding mass ratio (*m*_TX_:*m*_A336_) is 38.6.

**Figure 8:**
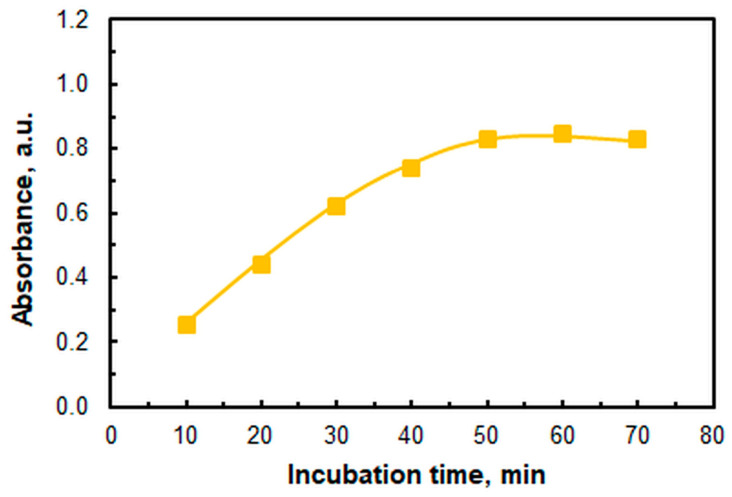
Effect of incubation time. *T* = 60 °C, *c*_Cu_ = 9.4 × 10^−6^ M, pH = 6, *w*_TX_ = 0.5%, *c*_A336_ = 3 × 10^−4^ M, *c*_4NC_ = 1.5 × 10^−4^ M, λ = 451 nm.

Figure 8 illustrates the relationship between absorbance and incubation time in a 60 °C water bath. The samples must remain at this temperature for at least 50 min. Therefore, the recommended incubation time is 50 min, starting from when the samples are submerged in preheated water. This incubation time is comparable to the times specified in other publications based on gravitational phase separation [29,51,52]. However, compared with other methods, the incubation time is often measured from the point at which the target temperature is reached, rather than from the moment of immersion.

The complete list of optimized parameters is shown in Table 1. The cooling time and SRP processing are based on the experiments described in previous papers [51,61]. As outlined in [52], the cooling process can be expedited by employing a centrifuge and an ice bath. However, the conventional method requires more labor and resources.

To obtain a reasonable blank absorbance, the mass of the diluted SRP was adjusted to 5.00 g. This mass included 0.5 mL of ethanol, which was added to decrease viscosity.

### 2.2. Formula and Extraction Constant

**Figure 9:**
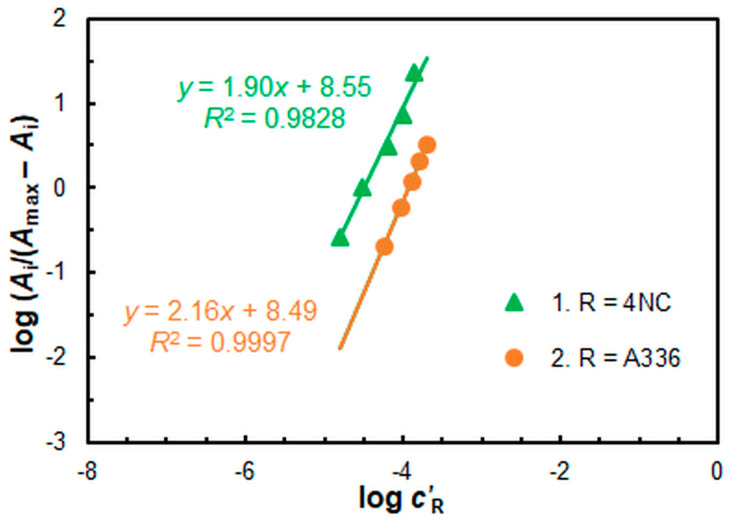
Determination of the 4NC:Cu^II^ (straight-line 1) and A336:Cu^II^ (straight-line 2) molar ratios via the mobile equilibrium method.

**Figure 10:**
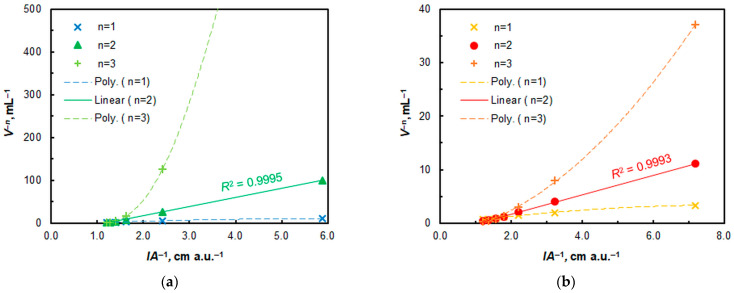
Determination of the 4NC:Cu^II^ (**a**) and A336:Cu^II^ (**b**) molar ratios using the Asmus method.

The presumed formula of the extracted ternary complex, (A336)_2_[CuL_2_], was verified by applying the mobile equilibrium method [62] (Figure 9) and the Asmus method [63] (Figure 10). Both methods revealed the following molar ratios between the components: 4NC:Cu = 2:1 and A336:Cu = 2:1. Consequently, the complex formation and CPE proceed according to Equation (2).

Cu^2+^_(aq)_ + 2 H_2_L_(aq)_ + 2 A336^+^_(aq)_ ≡ (A336)_2_[CuL_2_] _(SRP)_ + 4 H^+^ (2)

The extraction constant *K*_ex_ characterizing this equation was calculated using two methods: the mobile equilibrium method [62] and the Holme–Langmyhr method [64]. The results in logarithmic form are displayed in Table 2.

### 2.3. Analytical Characteristics

A calibration curve was constructed using Cu^II^ standards with concentrations (*γ*) ranging from 0.038 to 1.59 µg mL^−1^. The linear segment extended up to 0.763 μg mL^−1^ of Cu^II^ (*n* = 10; *R*^2^ = 0.9992), and the linear regression equation was *A* = 1.40*γ* − 0.005, where *A* is the absorbance. The standard deviations of the slope and intercept were determined to be 0.015 and 0.0060, respectively. The apparent molar absorption coefficient and Sandell sensitivity were (8.9 ± 0.1) × 10^4^ M^−1^ cm^−1^ and 0.71 ng cm^−2^, respectively. The calculated values for the limits of detection (LOD) and quantitation (LOQ) were 9.7 and 32 ng mL^−1^, respectively. These values were obtained by dividing the standard deviation of the blank (*n* = 10) by the slope, and then multiplying by three or ten. The preconcentration factor of 9.88 was determined by dividing the maximum initial volume (50 mL) by the volume of the final phase after dilution to 5.00 g (*V* = 5.06 mL). The volume of the final phase was derived by measuring its density with a pycnometer (*ρ* = 0.9877 g cm^−3^).

### 2.4. Effects of Diverse Ions and Analytical Applications

The effects of various ions are summarized in Table 3. The studied extraction–chromogenic system demonstrates significant resistance to high concentrations of alkali and halogen ions, including K^+^, Na^+^, Li^+^, Cl^−^, Br^−^, F^−^, and I^−^. It also tolerates substantial amounts of common acidic anions like NO_3_^−^ and SO_4_^2−^, enabling the dissolution and subsequent analysis of a wide range of samples. The most significant interference arises from V^V^, Mo^VI^, W^VI^, Cr^III^, and Fe^III^. These ions form stable, extractable complexes with 4NC [51,52,65,66], which can markedly increase the level of absorption. According to Refs. [54,67], the Cu^II^–4NC complex is more stable than the corresponding complexes of Zn^II^, Ni^II^, Co^II^, and Mg^II^—a finding that aligns with the current experiments. In addition, Mn^II^, Re^VII^, Cd^II^, and several other ions have shown satisfactory levels of tolerability. Given that many of these metals are the principal constituents of copper alloys [4], the developed method proves suitable for such samples.

In addition to artificial mixtures (Table 4), the novel method was tested in the analysis of the copper-containing dietary supplement Biligo 2 Plantis^®^ Copper (Artesania Agricola, Barcelona, Spain). According to the manufacturer, each ampoule of the product contains 858 µg Cu^II^, a dose intended to provide maximum benefits without the risk of excessive intake [68]. After dilution with water in a 100 mL flask, the product was analyzed for two days (five replicates per day). The results showed that the measured Cu^II^ content was statistically indistinguishable from the value specified by the manufacturer. The relative standard deviations were 2.4% on Day 1 and 3.2% on Day 2. The corresponding Cu^II^ masses were 870 µg (*R* = 101.4%) and 842 µg (*R* = 97.9%), respectively. A two-sample *t*-test comparing these measurements revealed no significant difference between the Day 1 and Day 2 results at the 95% confidence level (*t*_calculated_ = 1.84 < *t*_critical_ = 2.306).

The scope of the research was expanded to include an analysis of a solution for infusion of 0.9% NaCl (B Braun Melsungen AG, Melsungen, Germany), which is expected to contain no copper. The analysis was executed using the standard addition method at three levels between 38 ng/mL and 153 ng/mL (with four replicates at each level). The recoveries obtained were in the range of 91.9–102.6%, and the *y*-intercept of the straight line (*R*^2^ = 0.9994) was *b* = 0.0009 ± 0.0016.

### 2.5. Comparisons with Existing Methods and Evaluations with Modern Assessment Tools

Table 5 compares the key characteristics of the proposed method with those of other spectrophotometric CPE techniques. The current method distinguishes itself by its feasibility, practicality, simplicity, convenience, cost-effectiveness, environmental friendliness, and analytical competitiveness. Unlike conventional CPE methods [16,17,18,19,20,21,22,23,25,26,27,28,30,31,32,33,34,35,36,37,38,39,41,42,44], it eliminates the need for centrifugation by relying on spontaneous gravitational phase separation. In contrast to techniques that require pipettes, syringes, or other tools for final phase isolation [19,21,25,33,34,39,40,44], this approach enables faster and easier decantation. Additionally, unlike the RS-CPE method, it avoids the use of large volumes of organic solvents, [22]. The reagents used—4NC and A336—are inexpensive and readily available. Unlike other approaches that involve the synthesis of reagents, composites, or supramolecular systems [16,17,19,20,23,26,33,35,38,43], this method does not require any synthetic steps. All solutions are aqueous and exhibit long-term stability, eliminating the need for daily preparation, as required in the pyrogallol method [27].

The method provides a strongly linear calibration curve with stable absorbance over time and shows good tolerance to common ions and salt solutions. However, it has some limitations, including a relatively long analysis time, limited selectivity for certain ions, relatively high blank absorbance, and a lack of miniaturization and automation.

**Figure 11:**
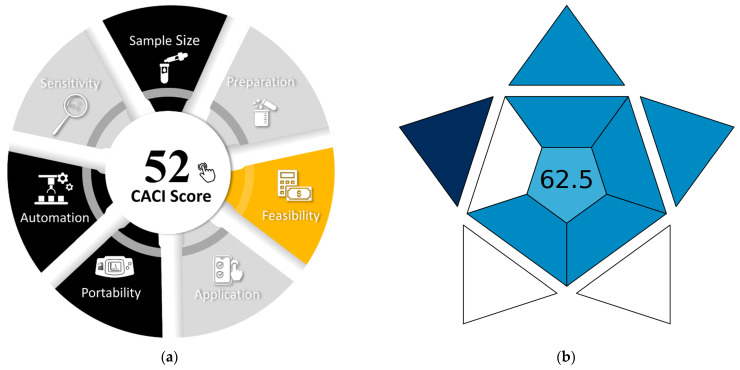
Application of CACI (**a**) and BAGI (**b**) for evaluating the present procedure.

Despite these drawbacks, the method achieves high scores on two recently developed assessment tools, the Blue Applicability Grade Index (BAGI) [69] and the Click Analytical Chemistry Index (CACI) [70], highlighting its practical applicability (Figure 11).

## 3. Materials and Methods

### 3.1. Reagents and Chemicals

The chemicals were obtained from Merck (Schnelldorf, Germany) and utilized without additional purification. The stock solution of Cu^II^ was prepared from CuSO_4_·5H_2_O at a concentration of 1 mg mL^−1^ [71], and the working solutions were properly diluted with water. In the optimization step, an aqueous solution of the nonionic surfactant TX at a mass fraction of 10% and a methanol solution of A336 at a concentration of 1 × 10^−2^ M were utilized. A mixed aqueous solution of these two substances was also used: it was prepared by combining 50 g of TX, 1.296 g of A336, and water in a 500 mL volumetric flask. The aqueous solutions of 4NC (≥96.0%) and NaNO_3_ had concentrations of 7.5 × 10^−3^ M and 1.3 M, respectively. Buffer solutions with pH ranging from 4.5 to 8.0 were prepared from 2.0 M solutions of NH_3_ and CH_3_COOH. The water was purified through either deionization (18.2 MΩ cm) or distillation.

### 3.2. Instrumentation

The UV–Vis spectrophotometers employed in this study were Ultrospec 3300 pro (Biochrom Ltd., Garforth, UK) and Drawell DU-8800RS (Drawell, Chongqing, China). Both instruments were equipped with 2.5 mL, 10 mm macro cuvettes. The CPE experiments were carried out using an Ohaus Pioneer PA214C analytical balance (Pubcompare, Parsippany, NJ, USA) and a GFL 1023 water bath (Gemini BV, Berlin, Germany). The pH measurements were taken using a WTW InoLab 7110 pH meter (Xylem Analytics, Weilheim, Germany).

### 3.3. Samples and Sample Preparation

The solution for infusion (0.9% NaCl, 500 mL; B Braun Melsungen AG, Melsungen, Germany) and the dietary supplement (Biligo 2 Plantis^®^ Copper, 20 ampoules for drinking 2 mL each; Artesania Agricola, Barcelona, Spain) were procured from a local pharmacy. The solution for infusion was applied without further processing. Aliquots of 5 mL were used for the analysis. The ampoules of the dietary supplement required appropriate dilution. For this purpose, one ampoule was diluted with water to 100 mL, and 1.8 mL aliquots was taken for analysis. To assess the effect of time on method characteristics, the analysis was performed on two consecutive days.

Artificial mixtures that mimic alloys were prepared from copper sulfate (15.3 µg Cu^II^) and corresponding amounts of salts of the other metals. The formulas of these salts are shown in Table 3.

### 3.4. Optimization Procedure

Solutions of TX, A336, Cu^II^, buffer, 4NC, and NaNO_3_ were transferred to 50 mL centrifuge tubes. The mixtures were diluted to 50 mL with water and then incubated in a water bath at 60 °C for 10–70 min. After a brief cooling period in running water, the tubes were stored in a freezer set at −20 °C for 50–60 min. This cooling process is essential for the solidification of SRPs, which can then be easily separated through decantation. Subsequently, 0.5 mL of C_2_H_5_OH and a few drops of water were added to the remaining SRPs, yielding a total mass of 5.00 g per sample. Finally, the contents were homogenized by shaking and loaded into cuvettes to measure the light absorbances.

### 3.5. Recommended Analytical Procedure

Place an aliquot of the analyzed solution containing 32–763 ng mL^−1^ Cu^II^ in a 50 mL centrifuge tube. Then, add 2.5 mL of the combined TX–A336 solution, 1 mL of the NaNO_3_ solution (*c* = 1.3 M), 1 mL of the pH 6 buffer solution, and 1 mL of the 4NC solution (*c* = 7.5 × 10^−3^ M). Dilute the sample to 50 mL with water. Place the tube in a water bath at 60 °C. After 50 min, cool the tube briefly under running water. Then, place it in a freezer set at −20 °C for 50 min. Decant the top liquid phase. Add 0.5 mL of C_2_H_5_OH to the SRP, and then add water until the total mass reaches 5.00 g. Homogenize the mixture by shaking and then load the cuvette. Measure the absorbance at *λ*_max_ = 451 nm against a blank prepared simultaneously. Finally, determine the unknown Cu^II^ concentration using a calibration or standard addition curve.

## 4. Conclusions

The formation of a ternary complex between Cu^II^ and 4NC in the presence of A336 was systematically investigated. The stoichiometry of the complex was determined, and the optimal conditions for a centrifuge-less extraction process were successfully established. This novel method can be categorized as a mixed micelle-mediated CL-CPE method, utilizing the synergistic properties of two surfactants: Triton X-114 and A336. This approach offers key benefits, including simplicity and efficiency, making it a promising alternative for the determination of copper(II).

Among the simplifications of the experimental procedure outlined in this paper that may positively impact future research is the use of a combined surfactant solution (A336 + TX). It is noteworthy that A336 is not soluble in water; however, it is soluble in an aqueous solution of TX114. The elimination of the need for an organic solvent to dissolve A336 renders the procedure more environmentally friendly. Moreover, the number of flasks, experimental steps, and costs is reduced.

The study of 4NC complexes is both interesting and promising. Building on the findings of this work, future research could explore the complexation behavior of iron and chromium ions under similar conditions. Such investigations would help address the challenge of determining Cu^II^ in the presence of these ions and further broaden the applicability of the proposed method.

## Figures and Tables

**Table 1:** The results of the system’s optimization.

Parameter	Optimal Value
Wavelength, nm	451
pH	6.0
Volume of the buffer, mL	1.0
Mass fraction of TX (*w*_TX_), %	0.5
Concentration of A336 (*c*_A336_), M	3.0 × 10^−4^
Concentration of 4NC (*c*_4NC_), M	1.5 × 10^−4^
Concentration of NaNO_3_ (*c*_NaNO3_), M	2.6 × 10^−2^
Incubation time at 60 °C (*t*_inc_), min	50
Refrigeration time (−20 °C), min	50
Test tube volume, mL	50
Mass * of the diluted SRP, g	5.00

* Diluted with ethanol (0.5 mL) and water.

**Table 2:** Conditional extraction constant values.

Method	Log *K*_ex_ *
Mobile equilibrium method	7.85 ± 0.08 (*n* = 5)
Holme–Langmyhr method	7.93 ± 0.11 (*n* = 4)

* ± Standard deviation (SD).

**Table 3:** Effect of diverse ions on the determination of 15.3 μg Cu^II^.

Ion	Added Salt Formula	Ion:Cu^II^ Mass Ratio	Cu^II^ Found, μg	*R*, %
Al^III^	Al_2_(SO_4_)_3_·18H_2_O	20 ^a^	16.0	105
Br^−^	NaBr	2000 ^b^	14.5	95.1
Ca^II^	Ca(NO_3_)_2_	500	14.6	95.6
Cd^II^	Cd(NO_3_)_2_·4H_2_O	250	14.7	96.5
Cl^−^	NaCl	2000 ^b^	14.9	97.6
Co^II^	Co(NO_3_)_2_·6H_2_O	200	15.1	99.3
Cr^III^	Cr_2_(SO_4_)_3_	0.1	16.2	106
F^−^	NaF	1000	15.9	104
Fe^III^	NH_4_Fe(SO_4_)_2_·12H_2_O	0.5	46.3	303
Hg^I^	Hg_2_(NO_3_)_2_	2	14.6	95.4
I^−^	KI	1000	15.3	100
Li^I^	LiCl	2000 ^b^	15.8	104
Mg^II^	MgSO_4_·7H_2_O	2000 ^b^	15.6	102
Mn^II^	MnSO_4_·5H_2_O	250 ^b^	15.3	100
Ni^II^	NiSO_4_·6H_2_O	250	15.0	98.6
Pb^II^	Pb(NO_3_)_2_	2	16.0	105
Re^VII^	NH_4_ReO_4_	250	14.7	96.2
V^V^	NH_4_VO_3_	0.2	23.0	148
W^VI^	Na_2_WO_4_·2H_2_O	1	17.8	117
Zn^II^	ZnSO_4_·7H_2_O	100	15.2	98.9

^a^ In the presence of F^−^ (7.7 mg). ^b^ Higher mass ratios were not studied.

**Table 4:** Analysis of artificial mixtures that simulate copper alloys.

Alloy	Ingredients [4]	Copper Found, %	RSD, %	*R*, %
Constantan	55.0% Cu, 45.0% Ni	54.6	3.8	99.3
German silver (#1)	55.0% Cu, 18.0% Ni, 27.0% Zn	54.5	3.6	99.1
German silver (#2)	62.0% Cu, 10.0% Ni, 28.0% Zn	63.6	2.7	102.5
Die-casting brass	60.0% Cu, 39.5% Zn; 0.5% Al	58.6	2.3	97.7
Manganin	86.0% Cu,12.0% Mn, 2.0% Ni	85.9	2.3	99.9
Cupro-nickel (#1)	68.0% Cu, 30.0% Zn; 1.0% Fe; 1.0% Mn	69. 8	2.0	102.6
Cupro-nickel (#2)	88.0% Cu, 10.0% Zn; 1.5% Fe; 0.5% Mn	87.7	1.0	99.7

**Table 5:** Comparison with other CPE methods coupled with UV/Vis.

Reagent(s)	Extraction Technique	Extractant(s)	pH	λ_max_, nm	ε·10^−4^, M^−1^ cm^−1^	Linear Range, ng mL^−1^	LOD, ng mL^−1^	Sample	Ref.
15-Crown-5	CPE	TX-114	4	252	1.02	200–7000	100	Spinach, tomato sauce, green tea, and black tea	[21]
ATAP	CPE	TX-114	4.5	608	43.7	4.0–115	1.20	Food, water, and biological samples	[19]
BIDP + OMIAFP6	IL-CPE	TX-100	9.0	610	N. R.	0.3–600	0.1	Water, fruit, and vegetable samples	[16]
BMPH	SUPRAS-CPE	TX-114	2	570	6.3	0.2–700.0	0.1	Food and drinking water	[17]
BrPAA	CPE	TX-114	8	512	2.32	7–1500	5.9	Herbal plants	[20]
DDTC	RS-CPE	TX-100	12	435	N. R.	Up to 50	0.4	Water samples, defatted milk powder, tea	[22]
DHMPhB	RT-CPE	TX-100	4.5	540	N. R.	20–950	6	Water samples	[23]
HTAR	CL-CPE	TX-114	5.9	535	25.4	4.5–254	1.34	Water samples, saline solution for infusion	[24]
Isoleucine	CPE	TX-100	9.0	230	N. R.	10–1000	5	Food and water samples	[30]
PAR	M-CPE	TX-114	8.0	515	N. R.	20–100	9.8	Tap water	[25]
PG + ST	UA-CPE	TX-114	5.5	532	N. R.	2–300	0.6	Beverages	[27]
Poly (SMIm)-Tris-Fe_3_O_4_	UA-CPE	TX-114 (Cu^I^) CTAB (Cu^II^)	7.0 5.0	347	N. R.	0.3−150 (Cu^I^) 10−350 (Cu^II^)	0.093 3.03	Lichen and mushroom samples	[26]
SAO	CPE	TX-114	4.2	380	N. R.	500−16,000	103	Urine	[28]
4NC+ A336	IL-MM-CL-CPE	TX-114	6.0	451	8.9	32–763	9.7	Synthetic mixtures and pharmaceutical samples	This work

Abbreviations: ATAP, 2-amino-4-(m-tolylazo)pyridine-3-ol; BIDP, 2,2(1H-benzo[d]imidazole-1,2-diyl) diphenol; BMPH, benzyl mono(2-pyridyl) hydrazine; BrPAA, 6-(4-bromophenylazo) m-anisidine; CTAB, cetyltrimethylammonium bromide; DDTC, diethyldithiocarbamate; DHDPhB, 6,7-dihydroxy-2,4-diphenylbenzopyrylium chloride; HTAR, 6-hexyl-4-(2-thiazolylazo)-resorcinol; M-CPE, micro-CPE; N. R., not reported; OMIAFP6, 1-octyl-3-methylimidazolium hexafluorophosphate; PAR, 4-(2-pyridylazo)-resorcinol; PG, pyrogallol; RS-CPE, rapidly synergistic CPE; RT-CPE, room temperature CPE; SAO, salicylaldoxime; ST, Safranin T; TX-100, Triton X-100; TX-114, Triton X-114; Poly (SMIm)-Tris-Fe_3_O_4_, styrene–maleicimide copolymer functionalized with tris(2-hydroxymethyl)aminomethane and magnetite; SUPRAS, supramolecular solvent; UA-CPE, ultrasound assisted CPE.

## Data Availability

Data are contained within the article.
